# Supplementary material for: Fibre orientations in collagen-containing tissues revealed with computational scattered light imaging and polarimetric second harmonic generation microscopy
Source: Sci Rep. 2025 Dec 7;16:1047. doi: 10.1038/s41598-025-30627-9 (PMC12783134; doi:10.1038/s41598-025-30627-9)
Supplement: Supplementary file 1 — Supplementary Material 1 [file 41598_2025_30627_MOESM1_ESM.pdf]

SUPPLEMENTARY INFORMATION:

Fibre orientations in collagen-containing tissues revealed with computational scattered light imaging and polarimetric second harmonic generation microscopy

Loes Ettema[1,*], Viktoras Mažeika[2,3], Mehdi Alizadeh[2,4,5,6], Hamed Abbasi[1,7], Virginijus Barzda[2,4,5], Miriam Menzel[1,*]

1 Department of Imaging Physics, Faculty of Applied Sciences, Delft University of Technology, Delft, The Netherlands
2 Laser Research Center, Faculty of Physics, Vilnius University, Vilnius, Lithuania
3 Institute of Biosciences, Life Sciences Center, Vilnius University, Vilnius, Lithuania
4 Department of Chemical and Physical Sciences, University of Toronto Mississauga, Mississauga, Ontario, Canada
5 Department of Physics, University of Toronto, Toronto, Ontario, Canada
6 Department of Physics, University of Ottawa, Ottawa, Ontario, Canada
7 Center for Optical Diagnostics and Therapy, Department of Otorhinolaryngology and Head and Neck Surgery, Erasmus MC, University Medical Center Rotterdam, Rotterdam, The Netherlands

Corresponding authors: L.Ettema@tudelft.nl, M.Menzel@tudelft.nl

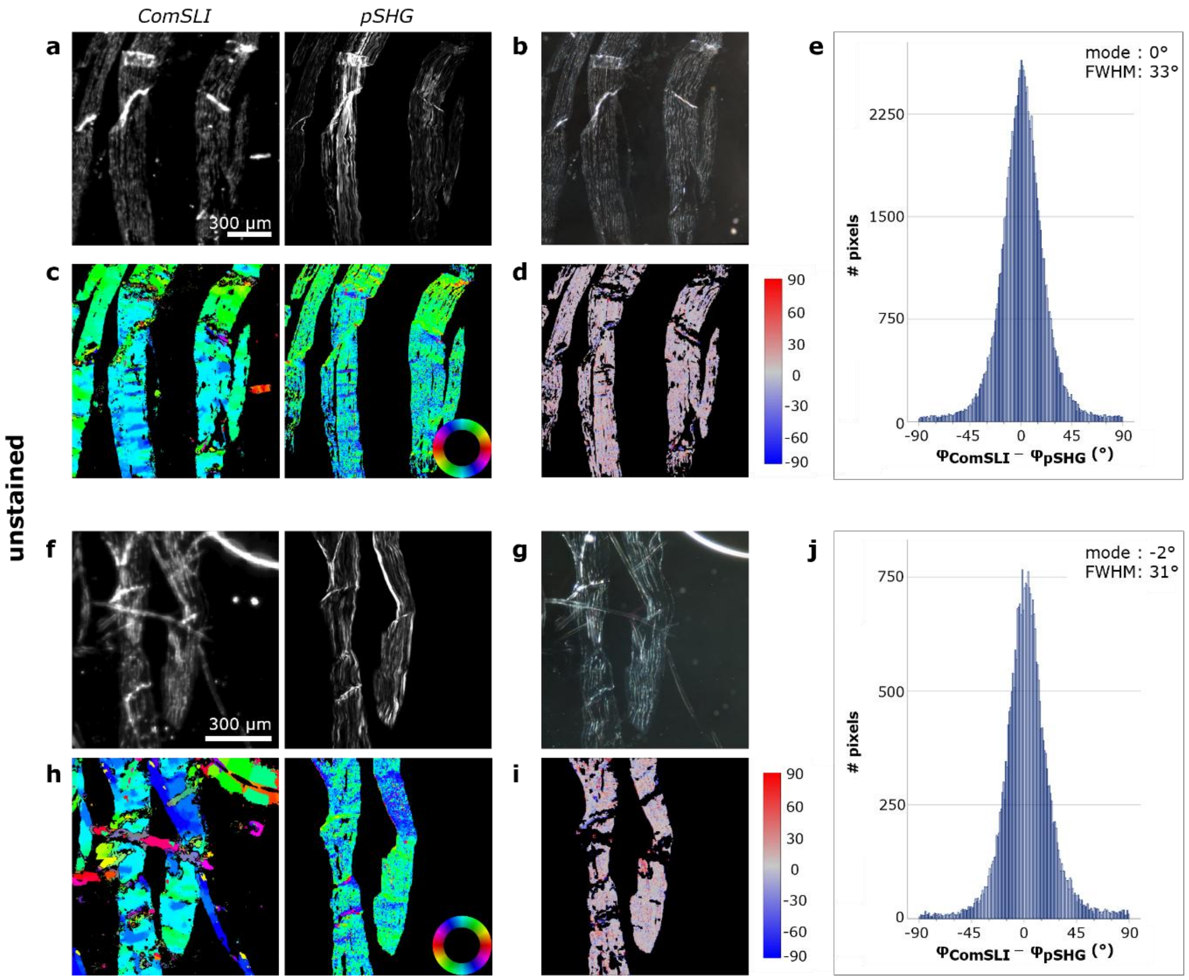

Supplementary Fig. 1. Comparison between ComSLI and pSHG-DSP on the in-plane fibre orientations obtained from unidirectional fibres in unstained rat tail tendon sections. (**a,f**) Left: Average scattering intensity maps from ComSLI. Right: Corresponding intensity maps from pSHG. (**b,g**) Corresponding dark-field images. (**c,h**) In-plane fibre orientation maps of ComSLI (left) and pSHG-DSP (right); orientations are indicated by different colours, see colour wheels. (**d,i**) Difference between the unidirectional fibre orientations obtained from ComSLI and pSHG-DSP (in degrees). (**e,j**) Corresponding histograms.

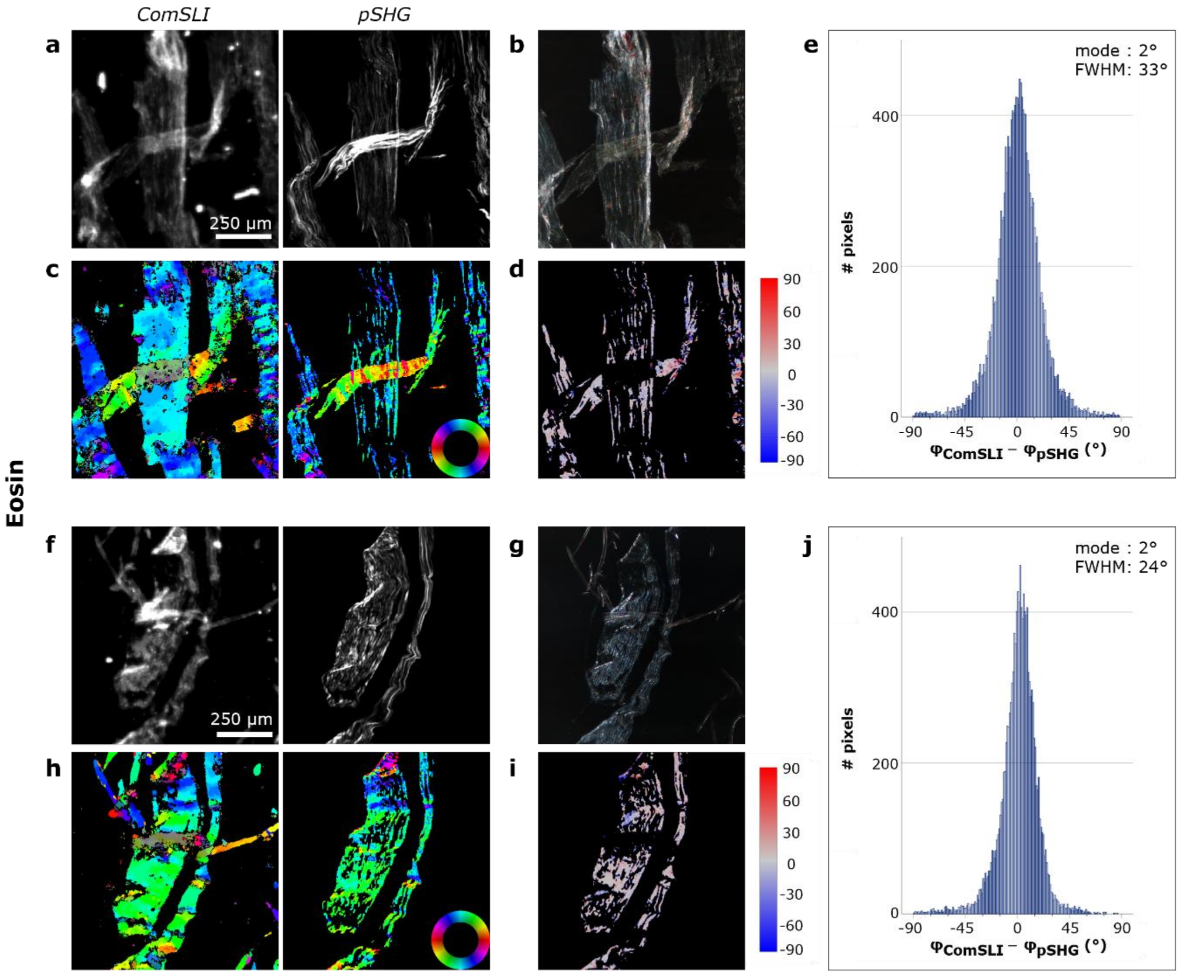

Supplementary Fig. 2. Comparison between ComSLI and pSHG-DSP on the in-plane fibre orientations obtained from unidirectional fibres in eosin-stained rat tail tendon sections. (**a,f**) Left: Average scattering intensity maps from ComSLI. Right: Corresponding intensity maps from pSHG. (**b,g**) Corresponding dark-field images. (**c,h**) In-plane fibre orientation maps of ComSLI (left) and pSHG-DSP (right); orientations are indicated by different colours; see colour wheels. (**d,i**) Difference between the unidirectional fibre orientations obtained from ComSLI and pSHG-DSP (in degrees). (**e,j**) Corresponding histograms.

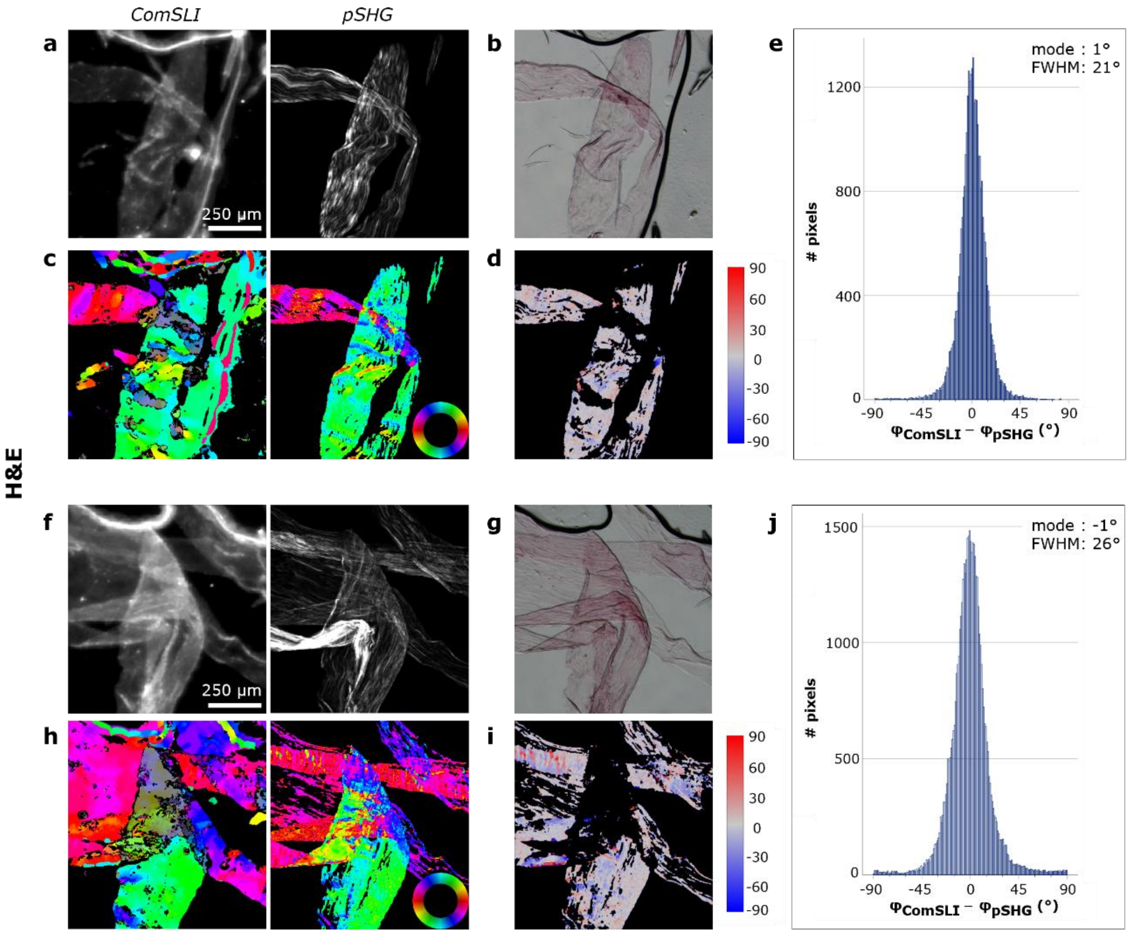

Supplementary Fig. 3. Comparison between ComSLI and pSHG-DSP on the in-plane fibre orientations obtained from unidirectional fibres in in-plane H&E-stained rat tail tendon sections. (**a,f**) Left: Average scattering intensity maps from ComSLI. Right: Corresponding intensity maps from pSHG. (**b,g**) Corresponding bright-field images. (**c,h**) In-plane fibre orientation maps of ComSLI (left) and pSHG-DSP (right); orientations are indicated by different colours; see colour wheels. (**d,i**) Difference between the unidirectional fibre orientation obtained from ComSLI and pSHG-DSP (in degrees). (**e,j**) Corresponding histograms.

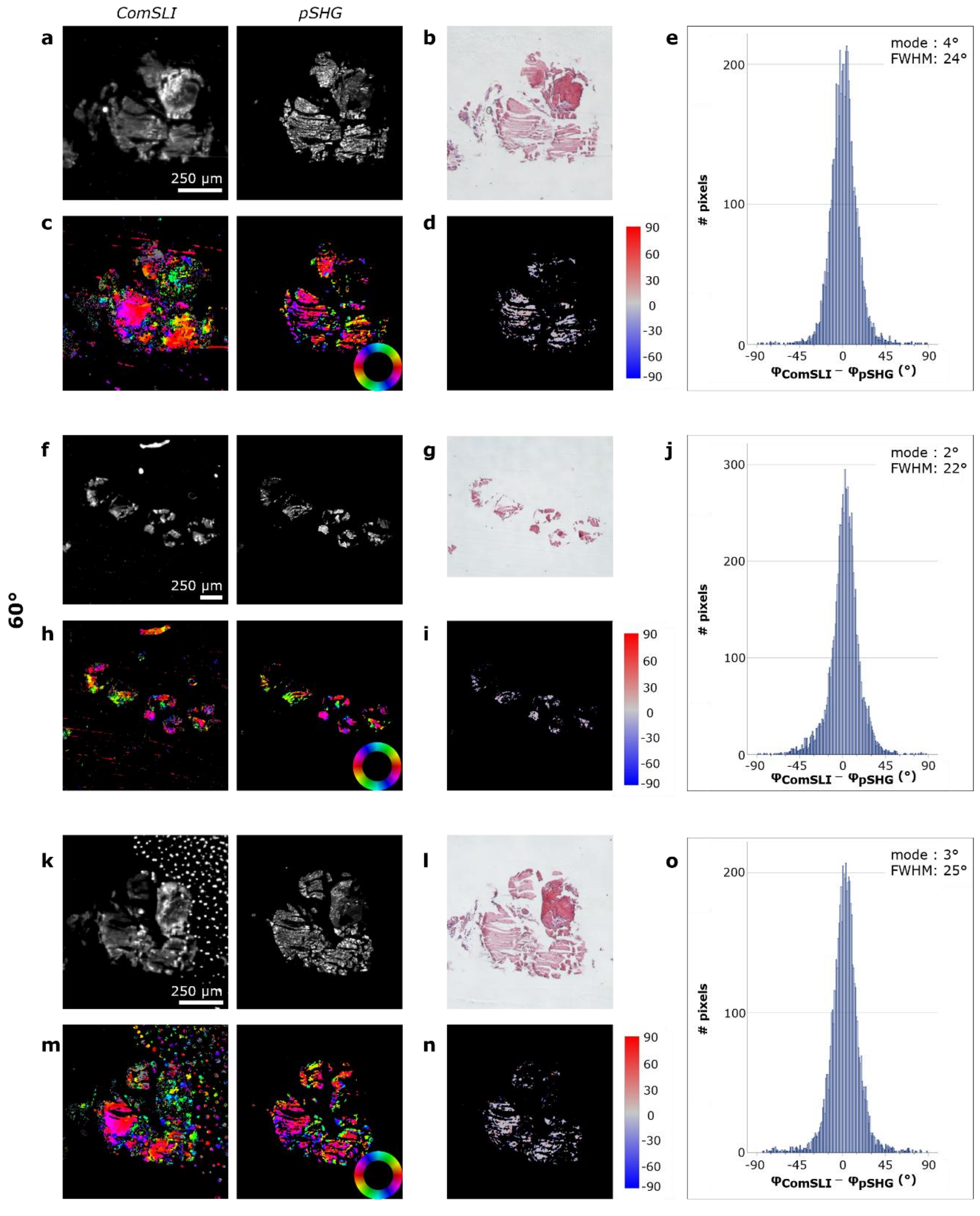

Supplementary Fig. 4. Comparison between ComSLI and pSHG-DSP on the in-plane fibre orientations obtained from 60°-cut rat tail tendon sections (H&E-stained). (**a,f,k**) Left: Average scattering intensity maps from ComSLI. Right: Corresponding intensity maps from pSHG. (**b,g,l**) Corresponding bright-field images. (**c,h,m**) In-plane fibre orientation maps of ComSLI (left) and pSHG-DSP (right); orientations are indicated by different colours; see colour wheels. (**d,i,n**) Difference between the unidirectional fibre orientations obtained from ComSLI and pSHG-DSP (in degrees). (**e,j,o**) Corresponding histograms.

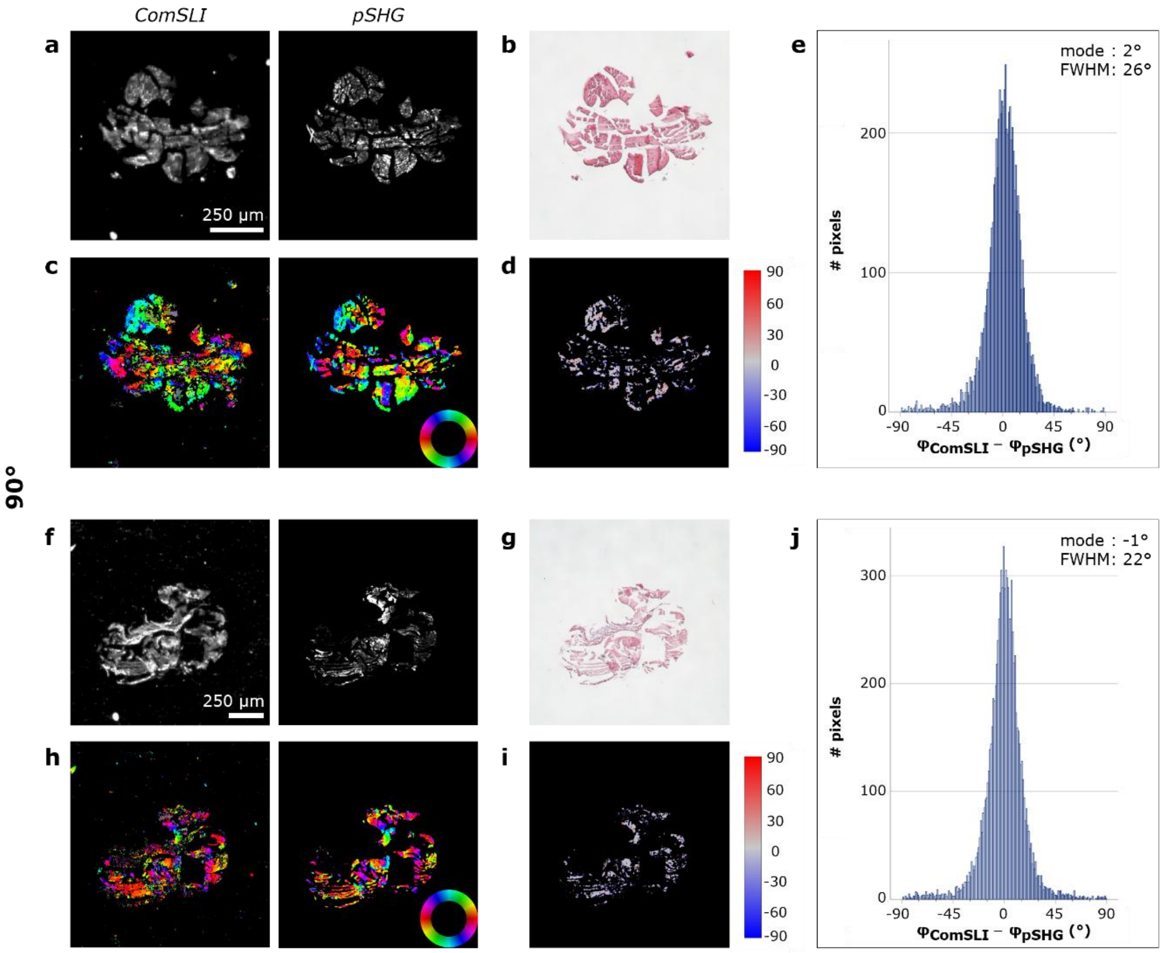

Supplementary Fig. 5. Comparison between ComSLI and pSHG-DSP on the in-plane fibre orientations obtained from 90°-cut rat tail tendon sections (H&E-stained). (**a,f**) Left: Average scattering intensity maps from ComSLI. Right: Corresponding intensity maps from pSHG. (**b,g**) Corresponding bright-field images. (**c,h**) In-plane fibre orientation maps of ComSLI (left) and pSHG-DSP (right); orientations are indicated by different colours; see colour wheels. (**d,i**) Difference between the unidirectional fibre orientations obtained from ComSLI and pSHG-DSP (in degrees). (**e,j**) Corresponding histograms.

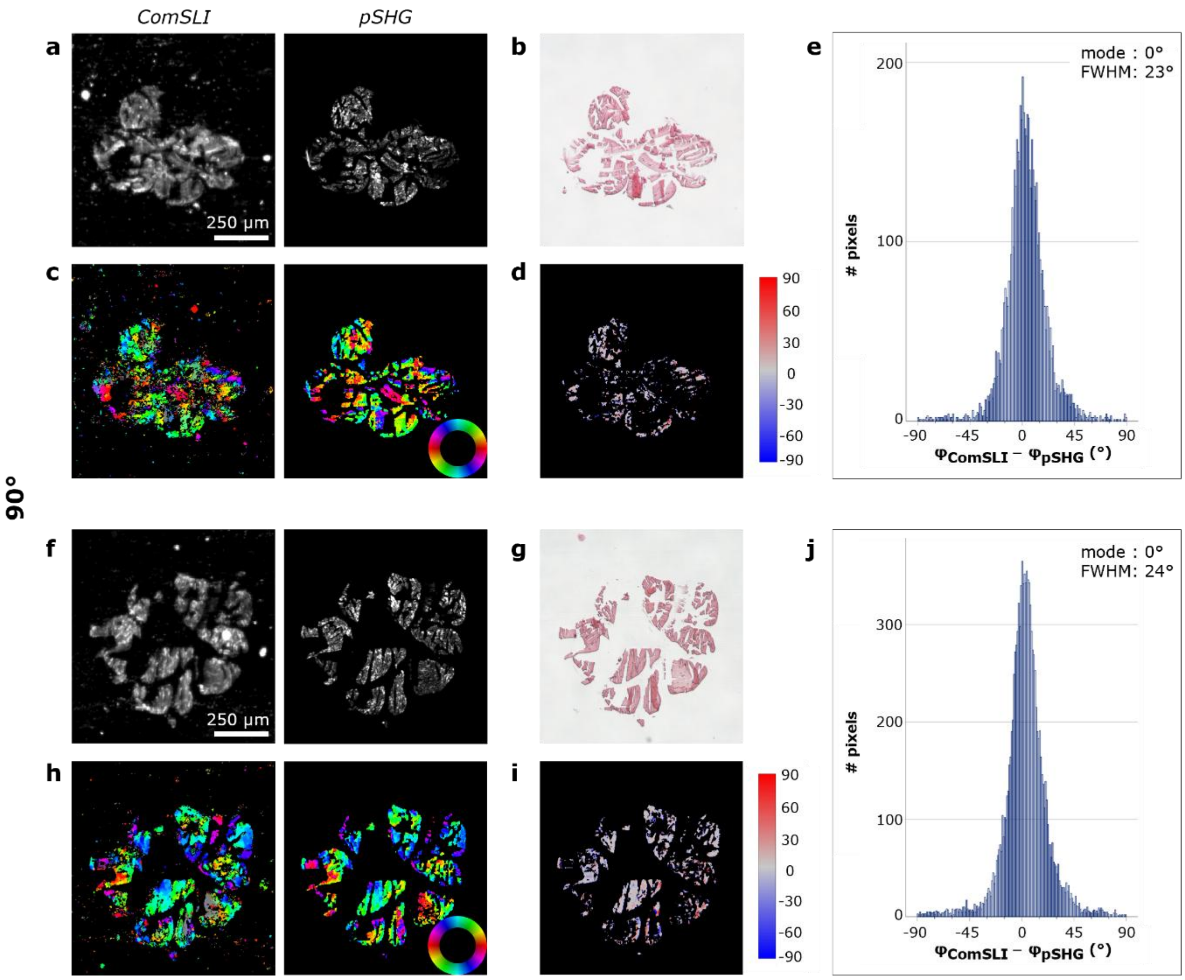

Supplementary Fig. 6. Comparison between ComSLI and pSHG-DSP on the in-plane fibre orientations obtained from 90°-cut rat tail tendon sections (H&E-stained). (**a,f**) Left: Average scattering intensity maps from ComSLI. Right: Corresponding intensity maps from pSHG. (**b,g**) Corresponding bright-field images. (**c,h**) In-plane fibre orientation maps of ComSLI (left) and pSHG-DSP (right); orientations are indicated by different colours; see colour wheels. (**d,i**) Difference between the unidirectional fibre orientations obtained from ComSLI and pSHG-DSP (in degrees). (**e,j**) Corresponding histograms.

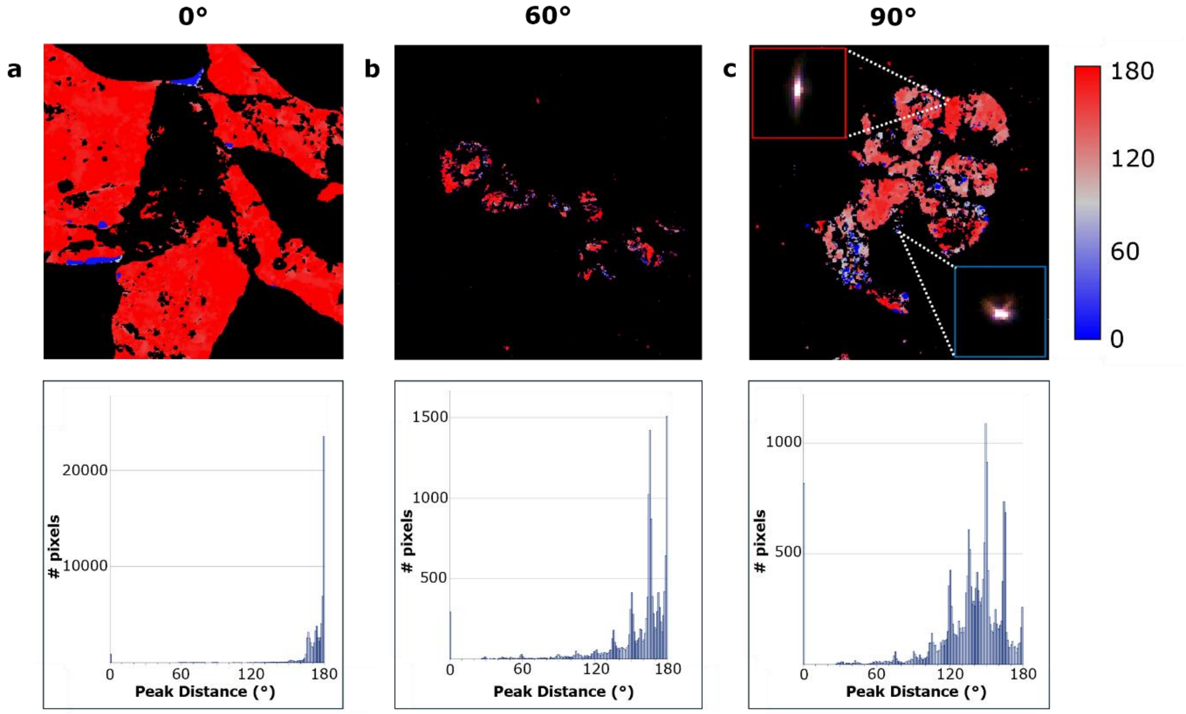

Supplementary Fig. 7. Peak distance maps and scattering patterns for rat tail tendon samples with different fibre inclination angles measured with ComSLI (H&E-stained). (**a**) Peak distance (°) map (top) for H&E-stained tail sections containing mostly in-plane fibre orientations and corresponding histogram (bottom) (mean = 171°); the peak distance map has been masked with the average scattering intensity map and the orientation map in order to get peak distances only for unidirectional fibres. (**b**) Peak distance (°) map (top) for an H&E-stained tail section obliquely cut under 60° and corresponding histogram (bottom) (mean = 155°). (**c**) Peak distance (°) map (top) for an H&E-stained tail section obliquely cut under 90° and corresponding histogram (bottom) (mean = 134°); the inserts show the scattering patterns of two image pixels: one corresponding to a pixel with a larger peak distance (red rectangle) and one corresponding to a pixel with smaller peak distance (blue rectangle), indicating more inclined fibres.
